# The Interaction of Selectins and PSGL-1 as a Key Component in Thrombus Formation and Cancer Progression

**DOI:** 10.1155/2017/6138145

**Published:** 2017-06-07

**Authors:** János Kappelmayer, Béla Nagy

**Affiliations:** Department of Laboratory Medicine, Faculty of Medicine, University of Debrecen, Debrecen, Hungary

## Abstract

Cellular interaction is inevitable in the pathomechanism of human disease. Formation of heterotypic cellular aggregates, between distinct cells of hematopoietic and nonhematopoietic origin, may be involved in events leading to inflammation and the complex process of cancer progression. Among adhesion receptors, the family of selectins with their ligands have been considered as one of the major contributors to cell-cell interactions. Consequently, the inhibition of the interplay between selectins and their ligands may have potential therapeutic benefits. In this review, we focus on the current evidence on the selectins as crucial modulators of inflammatory, thrombotic, and malignant disorders. Knowing that there is promiscuity in selectin binding, we outline the importance of a key protein that serves as a ligand for all selectins. This dimeric mucin, the P-selectin glycoprotein ligand 1 (PSGL-1), has emerged as a major player in inflammation, thrombus, and cancer development. We discuss the interaction of PSGL-1 with various selectins in physiological and pathological processes with particular emphasis on mechanisms that lead to severe disease.

## 1. Introduction

In the last three decades, our knowledge on the function of the receptor family of selectins and their ligands has been substantially extended in terms of the development and progression of several diseases, particularly inflammation, atherosclerosis, thrombosis, and malignancy. Furthermore, several mucins expressed on cancer cells and neutrophil extracellular traps (NETs) have been recently implicated to be involved in thrombosis and cancer development via selectin-mediated interaction. Due to substantial involvement of selectins and their counter-receptors in these serious conditions, they have become therapeutic targets in the prevention or at least alleviation of these disorders.

### 1.1. The Players and Their Nomenclature

Selectins obtained their names because of their ability to selectively bind carbohydrate moieties. Their ligands had long remained unidentified; nevertheless work in the 1980s led to the discovery of a dimeric mucin that is now uniformly designated as P-selectin glycoprotein ligand 1 (PSGL-1). The name PSGL-1 surmises that further molecules also exist as selectin ligands. Thus, the term PSGL-1 is misleading because of two reasons: (i) the mucin is not a glycoprotein ligand for P-selectin only but the major ligand for all three selectins and (ii) although it is true that there are several selectin binding proteins, the numbering of PSGL became obsolete as other selectin ligands are structurally different proteins. In the early years of selectin and PSGL-1 discovery, it was common that independent enthusiastic research groups investigated these phenomena in parallel using different approaches and the discoveries resulted in various nomenclatures on the very same proteins.

### 1.2. Selectin Structure and Function

There are three types of selectins that are all composed of the same domains and are distinguished from one another by their variable number of consensus repeats [1, 2]. The longest selectin molecule with 9 such motifs is P-selectin and it was named based on its first recognized source, the platelets. Subsequent studies also revealed that P-selectin is also detectable in endothelial cells; additionally these cells also possess a unique selectin naturally designated as E-selectin that is shorter in length compared to P-selectin as it contains 6 consensus repeats. The shortest selectin molecule, present in leukocytes, is L-selectin and contains only 2 consensus repeats [2]. There is considerable difference in the cellular appearance of these selectins not only in the sense that P-selectin is not cell-specific, as it is present on both platelets and endothelial cells, but also regarding its appearance in normal or activated cells [1, 2].

Research on selectins started with the identification of different monoclonal antibodies. Out of these antibodies one investigated in detail was the S12 clone [3]. It was found that nonactivated platelets do not react with S12, but following platelet activation by thrombin an intense labelling was obtained [3]. Studies revealed that this clone identifies a 140 kDa protein that is present in the alpha-granules of resting platelets and upon stimulation is expressed on the cell surface [4]. Thus, one of its designations was based on molecular mass; as such it was named granule membrane protein, GMP-140. Another group identified the same protein as a platelet activation dependent granule-external membrane protein [5]; as such it was designated as PADGEM [6]. Later it became evident that PADGEM, GMP-140, or P-selectin identifies the same cell surface molecule and subsequently obtained a number in the cluster of differentiation nomenclature as CD62P [2].

A major breakthrough was the discovery that, similarly to platelets, endothelial cells also contain a considerable amount of P-selectin. After its synthesis, this protein is transported to the Golgi apparatus where it is decorated with carbohydrates and finally transported to and stored in the Weibel-Palade bodies [7]. In addition to P-selectin, endothelial cells were also described to express a unique adhesion molecule, subsequently designated as E-selectin [8], but, unlike P-selectin, preformed E-selectin is not present in the endothelium. Thus, there is considerable difference in the kinetics of selectin expression in endothelial cells when stimulated, as P-selectin can be expressed on the endothelial surface within minutes, while E-selectin is de novo synthesized and a minimum of 2-3 hours are required for its surface expression.

The third member of the selectin family is L-selectin that was actually discovered earlier than the other two selectins [9] and is expressed on basically all types of leukocytes. It is different from the other two selectins not only in its size, but also in its expression as it is constitutively present on leukocytes [10]. Upon cell stimulation, their surface expression is usually downregulated. A short summary about the characteristics of selectins is shown in Table 1.

The discovery of the surface expression of selectins immediately implicated two important further areas of investigation. One that was plausible to be studied immediately upon their discovery was the identification of their soluble forms. The molecular mass of the soluble selectins is somewhat smaller than the membrane expressed forms, since they do not contain the transmembrane and intracellular domains. Shedding is the natural fate of surface-expressed receptors, for example, platelet CD40L, that is facilitated by proteolytic cleavage by (metallo)proteinases; nonetheless P-selectin shedding by this mechanism remains elusive [11]. Furthermore, PSGL-1 also regulates the rapid shedding and, in the absence of PSGL-1, P-selectin can be downregulated on activated platelets mostly by internalization [12]. In addition, a soluble form can also be released via the direct expression of its splice variant lacking the cytoplasmic domain [13]. It was shown earlier that, in addition to cell surface expression, the distribution and the intracellular trafficking of P-selectin are also important in leukocyte recruitment [14–16]. Several signaling molecules were described as regulators, and the internalization into clathrin-coated pits is also regulated by an endocytic receptor [17]. Similarly, the distribution of E-selectin in raft domains is also important for its adhesive capacity [18].

### 1.3. PSGL-1 Structure and Function

Another area of research has emerged from the arduous quest for the possible selectin ligands. Selectins interact weakly with small sialylated, fucosylated oligosaccharides, such as the tetrasaccharide sialyl-Lewis^x^, and they bind with higher affinity to glycans displayed on glycoproteins or proteoglycans. Thus, characterization of these high affinity selectin ligands was important, as they are key components in selectin-mediated leukocyte adhesion during inflammation. Although there are several glycosylated proteins that are capable of binding one particular selectin type, only one protein has emerged as the best characterized ligand for all three selectins. In the early 1990s, work from the laboratory of Dr. McEver described that endothelial cell P-selectin attaches to neutrophil glycoprotein via a lectin-like interaction [19]. This glycoprotein that was later proved to be the main counter receptor for selectins is a heavily glycosylated protein, where sialyl-Lewis^x^ is a necessary partner for the three selectins that bind the platelets, leukocytes, and endothelial cells. It is a 120 kDa homodimeric mucin that is a type I membrane protein consisting of 402 amino acids. The expression of the native protein would be insufficient for selectin binding, as PSGL-1 needs to be posttranslationally modified by sialic acid and fucose exclusively on O-glycans and to be sulfated on tyrosine residues to become functional. The importance of these posttranslational modifications is exemplified by the inability of lymphocyte PSGL-1 to bind to P-selectin. Although most lymphocytes express PSGL-1, only 10–20% actually bind P-selectin [20]. The necessity of PSGL-1 in selectin binding is interesting as it only represents a small fraction of the total sialyl-Lewis^x^ containing residues on the leukocyte surface and the copy number of this molecule is also relatively low [21].

**Figure 1:**
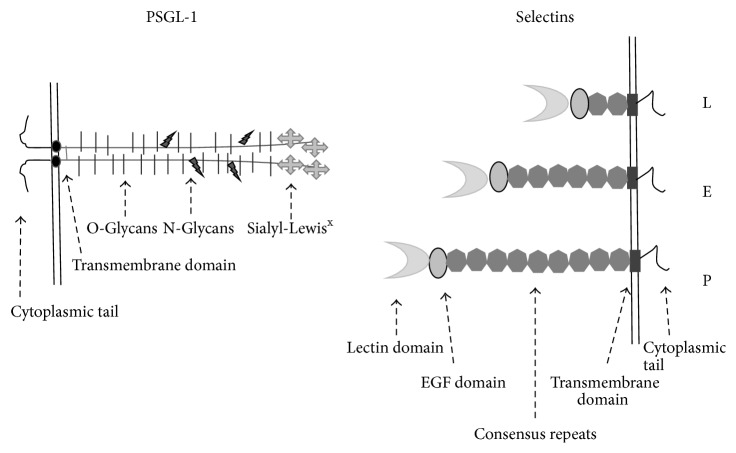
Structural features of selectins and PSGL-1.

Unlike P- and E-selectin but similarly to L-selectin, PSGL-1 is a constitutively expressed molecule on the surface of several hematopoietic and in some nonhematopoietic cells [22]. In circulating blood under flow conditions, selectins mediate the first adhesive step that is characterized by tethering and rolling of leukocytes on endothelial cells, platelets, or other leukocytes. L-selectin expressed on most leukocytes binds to ligands on endothelial cells and on other leukocytes; however, these molecules like the peripheral node addressins and the CD34 family of transmembrane sialomucins, such as podocalyxin, are not a part of this review. E-selectin expressed on activated endothelial cells also binds to ligands on most leukocytes and binds to activated platelets. There is a cellular network during inflammation that becomes complete by the participation of P-selectin on activated platelets and endothelial cells. The regulated expression of the selectins and their ligands initiates the inflammatory response and prepares the stage for a firm integrin-mediated leukocyte binding of the slowed leukocytes. It has been found that the inappropriate expression of these molecules contributes to leukocyte-mediated tissue damage in a variety of inflammatory and thrombotic disorders recently reviewed by Nagy Jr. et al. [23]. The structure of selectins and PSGL-1 is depicted in Figure 1.

### 1.4. PSGL-1 and Selectin Gene Polymorphisms

The functional effects of different individual or complex polymorphisms of selectins and PSGL-1 have been established in the development of vascular and metabolic diseases. PSGL-1 resembles the adhesive platelet receptor glycoprotein Ib alpha (GPIb*α*), as both proteins mediate the attachment of blood cells from blood. GPIb*α* is known to be polymorphic, so it was anticipated that PSGL-1 may also be polymorphic. Indeed, there is a relatively common genetic variation in PSGL-1 with variable number of tandem repeats (VNTR) affecting the length of the extracellular domain of PSGL-1 molecule via the distance from the P-selectin binding site to the cell surface [24]. Three allelic variants were identified in the human population. The 3 alleles, A, B, and C, from largest to smallest, contained 16, 15, and 14 decameric repeats, respectively, with the B variant lacking repeat 2 and the C variant retaining repeat 2 but lacking repeats 9 and 10. Allele frequencies were highest for the A variant and lowest for the C variant; the frequencies were described as 0.81, 0.17, and 0.02 in the white population. Homozygous carriers for the shorter B and C short alleles had lower risk for premature myocardial infarction due to lower adhesive capacity [25]. Further studies may clarify whether other haplotypes of P-selectin and/or PSGL-1 gene substantially influence the risk and outcomes of adverse vascular events with or without medication.

P-selectin is highly polymorphic having several genetic variants; the Thr715Pro variant located in the last consensus repeat region of P-selectin is probably the most intensively studied [26–30]. The substitution of threonine for proline induces a conformational change in the precursor protein, which may influence its intracellular transportation and secretion leading to reduced expression and/or shedding of P-selectin and thus fewer cellular interactions are developed. The Pro715 allele alters the kinetics of P-selectin release in patients with recurrent deep vein thrombosis [31]. The lectin domain of each selectin possesses the carbohydrate binding site and P-, E-, and L-selectin share 70% sequence identity over each of their three individual lectin domains. Changes in the amino acid sequence within the P- and E-selectin EGF domains have been shown to modulate the adherence of the proteins to sialyl-Lewis^x^ and heparin [32]. Another possibility by which polymorphisms may contribute to atherothrombotic processes is via the modulation of the release of soluble selectins. This has been shown for E-selectin as the Leu554Phe mutation results in a diminished soluble E-selectin release. Soluble E-selectin may be a protective factor in the progression of atherosclerosis as this may have a direct pathological consequence [33]. L-selectin is also polymorphic, but a recent large multiethnic analysis found that although the variants account for a significant level of soluble L-selectin variance, none of these variants were associated with clinical or subclinical cardiovascular disease [34].

## 2. Role of Selectins and PSGL-1 in Physiological Leukocyte Recruitment and during Inflammation

### 2.1. Classic Knowledge on Leukocyte Recruitment

The inflammatory process is accompanied by numerous molecular changes and several of these influence cell-cell interactions. Cellular interactions are extremely important also for the normal function of blood cells and the disturbance of this axis may lead to pathological states. As mentioned earlier the mere expression of adhesive proteins is not sufficient, the posttranslational modification of PSGL-1 is crucial for its function, and it is evidenced by the development of a human disease, the type II leukocyte adhesion deficiency (LAD-II). The affected patients have a mutation in the gene encoding a fucose transporter and thus cannot effectively incorporate fucose into selectin ligands [36]. As a result, leukocytes cannot bind any selectins and the patient suffers from bacterial infections of the mucosal membrane and the skin. A phenotype similar to that observed in LAD-II patients is detectable in mice lacking fucosyltransferase-7 [37].

If both P-selectin and PSGL-1 molecules are functional, they are the primary players in slowing down the leukocytes on the surface of activated endothelium resulting in the tethering and rolling of myeloid cells on inflamed endothelium. Endothelial P-selectin on the surface of activated endothelial cells and the constitutively expressed PSGL-1 are ideal molecules for capturing myeloid cells from the circulation as they are very long molecules extending far from the leukocyte surface and the endothelial surface layer. This process is delicately regulated by flow rate, P-selectin density, and receptor dimerization [38]. Each domain of the PSGL-1 molecule plays multiple roles in leukocyte rolling and extravasation, while the short cytoplasmic domain is dispensable for leukocyte rolling on P-selectin but is essential to activate *β*2 integrin to slow rolling on ICAM-1 [39].

Selectins interact with glycosaminoglycans and one practical consequence of this phenomenon was that unfractionated heparin is an inhibitor of selectin-PSGL-1 interactions. This anti-inflammatory effect of heparin was achieved at concentrations 10–50-fold lower than recommended for anticoagulation. It has also been suggested that low molecular weight heparins are much poorer inhibitors [40]. The inhibition occurs via blockade of P- and L-selectins and requires glucosamine 6-O-sulfation [41].

### 2.2. New Discoveries on How Leukocyte Recruitment Is Modulated

According to recent results, the leukocyte-endothelial bond strength is considerably influenced by the vessel diameter. If this diameter is comparable or smaller than that of the cell itself, the cells are not rolling as observed in venules but due to the small diameter they are travelling with a bullet motion. Under these circumstances the P-selectin-PSGL-1 interaction is not a weak interaction anymore but can provide a firm adherence to the wall of the capillary [42].

Another factor that may considerably influence physiological and pathological leukocyte recruitment is the variability of the endothelial surface layer in diverse anatomical locations. The majority of the experimental studies use the cremaster vasculature and observe neutrophil movement with microscopic techniques. Here the cell rolling and arrest occurs primarily in the postcapillary venules. Contrarily, in the lungs leukocyte extravasation occurs primarily through the capillaries and similarly in the kidneys the site of neutrophil extravasation is the glomerular capillaries and also in the liver the postcapillary venules have an inferior role in leukocyte diapedesis as it primarily happens in the liver sinusoids. These different anatomical locations may also display a large variability in the thickness of the endothelial surface layer that alters the pro- and antiadhesive properties of the endothelium [43].

But it is not only the endothelial cell that may exert variable contribution to the inflammatory process. A highly cited recent publication describes how neutrophils scan for activated platelets in the circulation to initiate inflammation [44]. By using intravital microscopy the authors elegantly demonstrated that the neutrophils recruited to inflamed vessels extend a PSGL-1 bearing microdomain into the vessel lumen that scans for activated platelets present in the bloodstream through P-selectin. The capacity of neutrophils to switch to a polarized morphology is essential as neutrophils that are unable to polarize or transduce signals through PSGL-1 display an aberrant crawling. Very recently studies have revealed that platelets and neutrophils have a mutual relationship. Platelets, in addition to their role in hemostatic processes, were found to have a considerable role in navigating leukocyte to their exit points in the inflamed microvasculature, as upon inflammation platelets were shown to immediately adhere at endothelial junctions in the smallest venular microvessels and capture neutrophils via CD40/CD40L dependent interactions. In this crosstalk, P-selectin-PSGL-1 ligation is crucial as it induces a conformational change of the surface-expressed leukocyte integrins. The blockade of this cellular partnership leads to misguided inefficient leukocyte responses leading to an ineffective leukocyte trafficking at the site of inflammation [45]. There are numerous further aspects in the modulation of leukocyte-platelet interactions that may not require the entire neutrophil. When activated platelets attach to neutrophils via P-selectin-PSGL-1 mediated binding, neutrophils extracellular vesicles (microvesicles or microparticles) are released that are involved in a multistep reciprocal crosstalk between platelets and neutrophils. These vesicles can be specifically internalized into platelets and subsequently relocated into intracellular platelet compartments enriched in cyclooxygenase that can process arachidonic acid into the vasoconstrictor and platelet aggregation promoter thromboxane A_2_ [46].

Finally there are several ways about how microorganisms may interfere with platelet-leukocyte interactions, thereby modulating the inflammatory reactions. One newer aspect of these effects was the recognition that extracellular fibrinogen binding protein (Efb) from* Staphylococcus aureus* inhibits the formation of platelet-leukocyte complexes via binding to P-selectin. Efb was shown to inhibit P-selectin-PSGL-1 interaction in both cell lysates and cell-free assays [47].

## 3. The Role of Selectins and PSGL-1 in Thrombus Development

### 3.1. Platelet and Soluble P-Selectin in Thrombosis

Clinically, P-selectin has been demonstrated to be a risk factor for recurrent venous thromboembolism. We have previously summarized several clinical studies where both platelet and soluble P-selectin were found to be elevated in patients with cardiovascular disorders [48]. Soluble and platelet P-selectin may not always alter their values in parallel. Activated platelets may be sequestered during thrombus formation and thus platelet P-selectin may underestimate the actual platelet activation. Activated platelets may also be attached to leukocytes; thus in a pioneering experimental work by Michelson, it was found that platelet-monocyte complexes are better markers for thrombotic tendency than platelet P-selectin that was formerly regarded as gold standard [49]. Furthermore, soluble P-selectin may be elevated disproportionately to platelet P-selectin because of the presence of selectin polymorphism that can influence P-selectin shedding [29]. On the other hand, although the major source of soluble P-selectin has undoubtedly been shown to be of platelet origin [50, 51], in certain situations endothelial cells as an alternative source have also been suggested [52].

### 3.2. PSGL-1 Mediated Cellular Interactions during Thrombus Formation

Abnormal neutrophil accumulation has been implicated in several inflammatory disorders like rheumatoid arthritis [53]. The persistent accumulation of neutrophils may lead to the release of elastase and toxic oxygen compounds that both potentiate tissue damage. The important role of the P-selectin-PSGL-1 axis in thrombus development has been demonstrated mostly in animal experiments. By using intravital microscopy, seminal discoveries were made mostly in the laboratory of Dr. Bruce Furie. Their experiments with real time intravital imaging revealed that the absence of P-selectin or PSGL-1 is accompanied by a deficiency of tissue factor accumulation and fibrin generation. On the microscopic images in mice they found that tissue factor antigen and fibrin accumulated within the first minute of vessel injury [54, 55]; however, leukocyte incorporation was not detectable in the developing thrombus in the first minutes. It was verified that circulating cellular microparticles include tissue factor associated with PSGL-1 and they observed that microparticles were captured by thrombus associated platelets through the interaction of microparticle PSGL-1 and P-selectin expressed by activated platelets [56, 57]. The importance of this axis was underlined in further murine studies that were carried out by the in vivo use of blocking antibodies to either adhesion molecule [58]. It was found that both antibodies abrogated lipopolysaccharide stimulation elicited platelet and leukocyte rolling and adhesion. Since platelets were also described to express functional PSGL-1 [59], it remained elusive whether this phenomenon occurs via platelet PSGL-1 or via the platelet-leukocyte binding.

The real time imaging methodology in the study of thrombus formation in mice has become a powerful technique that such studies were worth presented in a real time visualized form [60]. Based on the above results, the P-selectin-PSGL-1 axis is important for tissue factor induction and cell aggregate formation between platelets and leukocytes. The binding of these two adhesive proteins also leads to other alterations like the *β*2 integrin Mac-1 conformational change on the monocyte surface. In addition to these data, biologically relevant concentrations of soluble P-selectin were found to stimulate phosphatidylserine (PS) expression in a time and concentration dependent manner. The effect was already observed slightly above the reference range and was reaching its peak value when soluble P-selectin became 6-fold elevated [61]. The PS-inducing capability was observed with both soluble and platelet membrane-bound form of P-selectin and resulted in a surface-dependent thrombin generation on monocytes. As described in the previous chapter, microvesicles are very important in inflammation and thrombus initiation. These cellular fragments are a result of selective membrane shedding. It has been shown that monocyte/macrophage derived microvesicles are deficient in CD45 but are enriched in PSGL-1 and tissue factor. It was also found that lipid rafts are also rich in tissue factor and PSGL-1 but not CD45 and consistent with the raft origin of these tissue factor-bearing microvesicles their shedding was significantly reduced with depletion of the membrane cholesterol. The microvesicles may fuse with platelets transferring both proteins and lipids to the platelet membrane [62].

Several other groups have addressed the question of thrombus formation in relation to P-selectin ligation. The role in thrombus promotion has been verified and it was also observed by flow cytometry that platelet P-selectin initiates platelet aggregation by inducing microaggregate formation [63]. This process is somewhat similar to the role of L-selectin via its interaction with PSGL-1, where it initiates the aggregation process and increases the *β*2 integrin affinity and avidity for its ligands in neutrophils [64]. If appropriate posttranslational modifications occur and PSGL-1 becomes functional, the expression rate of the protein may become a pathogenic factor. By investigating monocyte subsets in patients suffering from various cardiovascular disorders, it was found that the expression of PSGL-1 on CD14^++^/CD16^+^ monocytes is significantly increased in patients with unstable angina and acute myocardial infarction (AMI) compared to controls. Moreover the dramatic increase at the onset of AMI was decreased during the chronic phase and the intensity of PSGL-1 staining was significantly higher in patients with plaque rupture [65].

Since PSGL-1-selectin interactions are crucial to thrombus formation, it was plausible to hypothesize that the lack of PSGL-1 would be protective against thrombosis. Several studies were launched with this hypothesis and they unequivocally proved that the lack of this adhesive protein is indeed protective against induced arterial and venous thrombosis in murine models [66–68].

**Figure 2:**
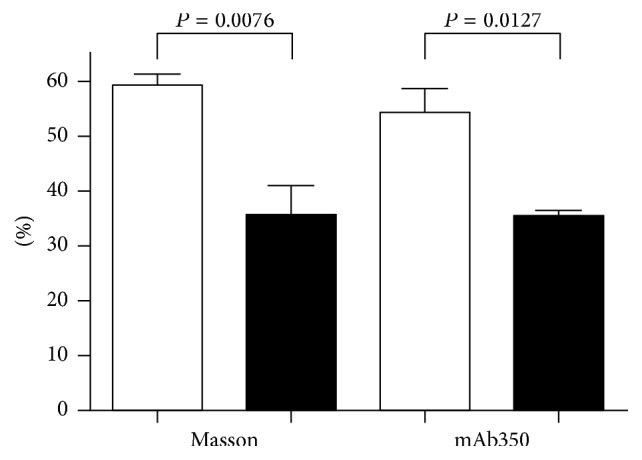
Ratio of vessels occluded with thrombi after collagen-epinephrine challenge in PSGL-1 knockout (filled bars) and wild-type (open bars) mice. A significantly reduced rate of thrombus formation was observed in knockout animals both with Masson trichrome staining and with an immunohistochemical staining to mouse fibrin* (reprinted from Thrombosis Research with permission by Elsevier).*

**Figure 3:**
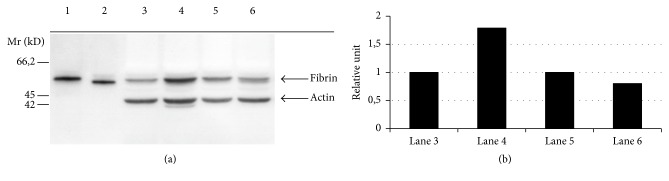
Quantity of fibrin deposition in the lungs. The western blot analysis of lung extracts shows remarkable difference. (a) Lane 1: fibrinogen standard, lane 2: fibrin standard, lane 3: wild-type mice treated with saline, lane 4: wild-type mice treated with collagen and epinephrine, lane 5: knockout mice treated with saline, and lane 6: knockout mice treated with collagen and epinephrine. (b) After thrombotic challenge, twice as much fibrin deposits were found in the wild-type mice compared to knockout animals* (reprinted from Thrombosis Research with permission by Elsevier).*

**Figure 4:**
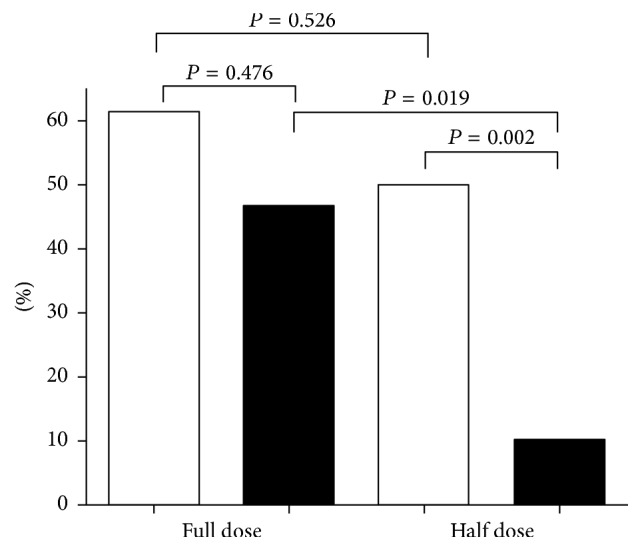
Survival rates of wild-type and knockout mice within 30 minutes after thrombotic challenge. The percentage of perished wild-type mice (open bars) and knockout mice (solid bars) after administration of full dose and half dose collagen + epinephrine (full dose: 15 *μ*g collagen + 3 *μ*g epinephrine, half dose: 7.5 *μ*g collagen + 1.5 *μ*g epinephrine)* (reprinted from Thrombosis Research with permission by Elsevier).*

If thrombotic stimuli are carefully chosen in these models, a difference in the number of vessels occluded with thrombi was evident whether determined by conventional or immunochemical staining (Figure 2); also less fibrin was deposited in the lungs of PSGL-1 knockout animals (Figure 3) that can partly be explained by the PS-inducing capacity of the P-selectin-PSGL-1 axis. All these effects contribute to a better survival rate in PSGL-1 knockout mice upon thrombotic challenge (Figure 4). The above data about the selectin-PSGL-1 connection served as examples for physiological function and as a pathogenic player in vascular disorders.

Nevertheless, there are few examples on the beneficial effects of the selectin-PSGL-1 interactions. As pointed out previously, the formation of microvesicles and their interaction with blood cells have been described as a pathognomic characteristic in thrombotic disorders. However, the presence of microvesicles may become beneficial and the vesicles that were generated by the P-selectin-PSGL-1 interaction corrected the hemorrhagic disorders as was described in an animal model of haemophilia [69]. More recently, it was also shown that, after laser-induced vascular injury in mice, neutrophil granulocytes recruited endothelial colony-forming cells at the site of vascular injury via a PSGL-1-L-selectin interaction and via this effect contributed to angiogenesis and the regeneration of the injured vessel [70].

## 4. The Role of the Selectin-PSGL-1 Axis in Malignancies

Similarly to inflammation and thrombus formation as discussed above, tumor growth and the development of metastasis comprise of a cascade of various cellular events regulated by a large number of adhesion molecules including selectins from the initial step up to the advanced stages of malignancy [71]. Basically, selectins and their ligands may be involved in cancer progression in two ways: (i) selectin ligands (mucins) are expressed on cancer cells to bind to selectins on the surface of (activated) normal blood cells or endothelial cells that facilitates the arrest and extravasation of tumor cells and (ii) in turn the tumor itself rarely expresses selectin to exploit these interactions above for aggregating with leukocytes and endothelial cells to seed distant metastases. Based on former investigations, there are three main approaches to analyze the role and the mechanism of selectin/selectin ligand pairing in cancer propagation: (i) the investigation of knockout mice lacking selectin or with deficiency of endogenous enzyme(s) involved in ligand expression in comparison to wild-type counterparts, (ii) the application of neutralizing antibodies against selectin(s) in animals to block these interactions in vivo to study altered tumorgenesis, and (iii) the utilization of these agents in tumor cell cultures among in vitro (flow) conditions to influence cellular interactions.

In respect to abnormal selectin ligand expression, malignant cells are characterized by mucins with abnormal glycosylation [72]. Selectins predominantly bind sialyl-Lewis^x^ or sialyl-Lewis^a^ fucosylated carbohydrate ligands on tumor cells, which are synthesized by different glycosyltransferases [73]. The increased function of fucosyltransferase-7 resulted in a higher sialyl-Lewis^x^ expression causing enhanced lung cancer progression [74]. These data have recently been supported by others when fucosyltransferase-7-deficient mice displayed a reduced recruitment of monocytes to metastasizing tumor cells correlated with attenuated metastasis [75]. Hence, the degree of abnormal glycosylation with altered expression of carbohydrate selectin ligands by cancer cells correlates with metastasis formation and poor prognosis for cancer patients [73]. The altered expression of sialyl-Lewis^x/a^ containing mucins allows tumor cells to interact with their microenvironment via binding to selectins on blood cells and endothelium that influences metastatic spread [76]. For example, mucin 16 overexpressed on pancreatic cancer cells bound to E- and L-selectin under flow [77]. Thus, mucin removal from tumor cells could effectively attenuate metastasis [78].

**Figure 5:**
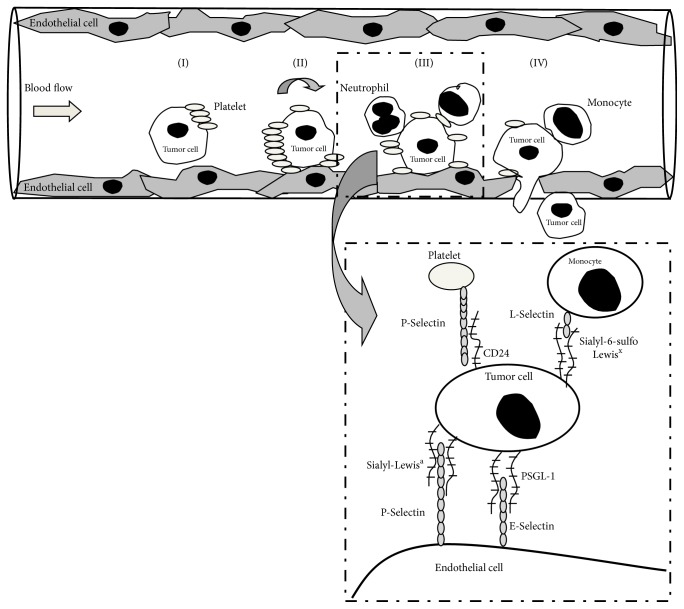
A schematic figure on the development of selectin-mediated heterotypic interactions among tumor cells, leukocytes, platelets, and endothelial cells during metastasis formation. Tumor cells circulate in the blood stream until they tether (I), roll (II), and then are arrested on endothelial cells (III) and finally migrate from the vasculature (IV). Activated platelets aggregate with cancer cells via P-selectin protecting them from innate immune system and permitting further leukocyte binding. Induced endothelial cell activation by tumor cells results in E- and P-selectin expression with additional recruitment of reactive neutrophils and monocytes to cancer cells regulated via L-selectin. As being magnified, selectin ligands (e.g., PSGL-1, sialyl-Lewis^x^) are expressed on malignant cells to bind selectins that are expressed on normal blood cells and endothelial cells. See details in the text. Of note, there are several other receptors and integrins involved in these interactions but that cannot be depicted here.

Hematogenous metastasis of cancer occurs in the later stage of tumor progression that is responsible for death in most cancer patients. After tumor cells entered the blood stream, they circulate and bind to (i) platelets, which support their extravasation and protect them from innate immune system and mechanical stress, (ii) leukocytes, which may support the adhesion of cancer cells to vessel wall, and (iii) endothelial cells to adhere and then migrate from the vasculature. First, after binding to activated platelets via P-selectin, tumor cells tether and then roll on the endothelial cells to be finally arrested in the microvasculature of distant organs. Due to the formation of these heterotypic aggregates, endothelial cell activation is induced resulting in enhanced E- and P-selectin expression. The recruitment of reactive neutrophils and monocytes to cancer cells is regulated via L-selectin as well as endothelial-mediated interactions [79, 80]. Subsequently, platelets secrete a number of bioactive mediators, such as platelet-derived growth factor (PDGF), vascular endothelial growth factor (VEGF), fibrinogen, and thrombospondin to provide mitogenic triggers for cancer [81]. This environment establishes first a “premetastatic niche” where the primary tumor cells can survive, proliferate, and later metastasize. Before the arrival of tumor cells, myeloid precursor cells are mobilized that express VEGF receptor 1 or tumor necrosis factor alpha to provide a permissive niche for migrating cancer [82, 83]. Developing niche is shaped by additional cellular interactions, especially through E-selectin between circulating tumor cells and endothelial cells during extravasation process [84]. Consequently, an E-selectin targeted aptamer could reduce hematogenous metastases of breast cancer in a mouse model [85]. Very recently, myeloid-derived suppressor cells have been shown to promote the arrest of cancer cells via IL-1*β*-mediated E-selectin expression on endothelial cells [86]. In turn, activated endothelium produces several chemokines (CCL5) to support the extravasation of cancer cells [71, 79, 87]. Attachment of tumor cells to endothelium is also promoted by innate immune cells; for example, neutrophils increased melanoma cell extravasation by IL-8 production [88]. Subsequently, PSGL-1 mediated recruitment of monocytes facilitates tumor extravasation [75]. These cellular events are summarized and depicted in Figure 5.

### 4.1. Role of P-Selectin during Tumor Progression

P-selectin was started to be widely investigated in relation to cancer biology after its role had been analyzed under inflammatory and thrombotic conditions [54, 89]. During the 1990s, the first reports were published demonstrating that P-selectin can bind to several human cancers [90–92]. As a result, P-selectin deficiency in mice caused attenuated human colon carcinoma growth and metastasis in vivo [93] and impaired murine adenocarcinoma progression [94]. The formation of distant metastases of small cell lung cancer (SCLC) was also significantly reduced when this cell line was xenografted into P-selectin knockout mice [95]. Substantial evidence demonstrated that platelets interact with circulating malignant cells to form tumor microemboli via P-selectin, and these events may help cancer cell arrest in distant organs where they adhere to vessels [81, 96]. Actually, the involvement of platelets in tumor progression was far earlier suggested based on those animal models, when pulmonary metastasis was inhibited in different types of cancers in the presence of induced thrombocytopenia or by the inhibition of fibronectin and von Willebrand factor with monoclonal antibodies [97]. The expression of sialyl-Lewis^a^, sialyl-Lewis^x^, or PSGL-1 was demonstrated on the surface of human melanoma [98, 99], breast cancer [100], and different SCLC cell lines [95, 101] to interact with P-selectin on platelets. PSGL-1 negative breast and SCLC cells may express O-glycosylated glycoprotein, CD24, which was found to be important in the dissemination of tumor cells [102]. The “platelet cloak” around tumor cells provides further interactions of monocytes to malignant cells via platelets [78] but also protects against natural killer- (NK-) mediated clearance of tumor cells [103]. Of note, P-selectin on endothelial cells additionally contributes to metastasis based on attenuated melanoma lung metastasis after the transplantation of bone marrow from P-selectin-deficient into wild-type mice [104]. Malignancies are often associated with inflammation, enhanced cytokine expression, and lymphocyte infiltration of tumor tissue that are more likely to facilitate growth and spread of cancer than being effective in a host antitumor response [105]. In breast cancer, the loss of P-selectin inhibited the infiltration of regulatory T-cells and reduced levels of proinflammatory cytokines, such as IL-4, IL-10, and TGF-*β*, were measured in the tumors resulting in a better survival ratio [106].

Selective inhibition of selectins may be potential therapeutic targets for preventing hematogenous metastasis. Interaction of cancer cells with platelets and endothelial cells via P-selectin can be blocked by unfractionated heparin in a clinically tolerable concentration range mimicking its ligands that reduce the organ colonization of cancer [104]. Recently, low molecular weight heparin, Tinzaparin, effectively blocked P-selectin in vivo reducing metastasis formation in a B16F10 melanoma mice model [107]. On the other hand, treatment of the tumor cells with O-sialoglycoproteinase prevented endothelial cell activation and chemokine (CCL5) production leading to decreased metastatic microenvironment [87]. Finally, enzymatic removal of sulfation of SM4 from the surface of MC-38 colon carcinoma cells resulted in decreased P-selectin binding on platelets with attenuated metastasis [108]. Overall, platelets with P-selectin are considered as the key enhancers of hematogenous dissemination, tumor survival, and tissue colonization [78], as they initiate the early phase of metastatic tissue colonization via P-selectin [71], while activated endothelium with increased P-selectin exposure supports these events [104].

### 4.2. Involvement of E-Selectin in Cancer Progression

E-selectin is an important adhesion receptor on activated endothelial cells for leukocytes, but cancer cell migration is also mediated by E-selectin to be arrested on microvasculature as one of the initiating events during metastasis [79]. Subsequently, endothelial cells become activated by the accumulation of malignant cells and P-selectin-dependent platelet-tumor cell interactions. Hence, they show increased expression of E-selectin with induced production of different chemokines and facilitate the subsequent recruitment of monocytes and myeloid cells [66]. The blocking of E-selectin function attenuated liver [109] and lung metastases by colon carcinoma cells [110]; however, others found E-selectin dispensable in lung metastasis indicating its role merely in local activation of lung microvascular endothelial cells [80]. E-selectin binding to colon cancer cells can alter gene expression to promote further metastases [111]. Moreover, signal transductions in endothelial cells are also triggered to regulate the integrity of the endothelial layer for transendothelial migration of colon tumor cells [112].

E-selectin ligands are mostly mucins. Increased expression of CD44 known as hematopoietic cell E-/L-selectin ligand (HCELL) on colon carcinoma resulted in an enhanced adherence to activated endothelium [113]. Additionally, human colon carcinoma cells can express other E-selectin ligands, such as death receptor-3, LAMP-1, and LAMP-2 that provide survival advantages for this malignancy [114, 115]. The recently discovered Mac-2 binding protein can be expressed by breast cancer cells to bind this selectin upon metastasis [116].

Heparin is an excellent inhibitor of P- and L-selectin binding to the sialyl-Lewis^x^, but no effect on E-selectin was observed [117]. In contrast, a bile acid acylated-heparin derivative showed an inhibitory effect on E- and P-selectin-mediated interactions reducing adhesion and invasion of B16F10 cells into the lung in mice [118]. When a soluble form of E-selectin, the recombinant fusion protein E-selectin-immunoglobulin, was used against E-selectin, this treatment impaired lung metastasis by colon carcinoma [110]. On the other hand, blocking its sialyl-Lewis^a^ ligand with a specific antibody [119] or a mimetic peptide (DLWDWVVGKPAG) [120] inhibited the dissemination of pancreatic cancer in the peritoneal cavity of nude mice and the metastasis formation of B16F10 melanoma cells to the lung, respectively.

### 4.3. Function of L-Selectin in Cancer Progression

L-selectin expression is restricted to neutrophils, monocytes, and natural killer (NK) cells that also display a role in regulating metastasis. First, it was shown that L-selectin facilitated the lymph node metastasis [121]. In contrast to the contribution of P-selectin positive platelets, leukocyte L-selectin can facilitate tumor metastasis at later stage, as L-selectin mediates the leukocyte recruitment to tumor emboli after P-selectin-mediated platelet-cancer cell aggregates are formed [71]. Thus, L-selectin deficiency does not affect initial tumor cell embolization, since the association of CD11b positive myeloid cells with tumor cells was reduced and tumor cell survival was diminished 24 hours later [122]. Consequently, the enhanced expression of L-selectin ligands in the endothelium and in the tumor emboli also correlates with leukocyte infiltration. In summary, L-selectin facilitates metastasis formation via leukocyte-endothelium interactions, which is supported by L-selectin ligand induction by fucosyltransferase-7 [122]. When the host response of inflammatory cell infiltration in malignant melanoma was investigated to see how this selectin regulated these events as an antitumor reaction, pulmonary metastasis was enhanced by the loss of L-selectin due to impaired migration of NK cells, CD4+ and CD8+ T-cells into the lung tissue; however, cytotoxic response was unaffected [123].

L-selectin has several ligands expressed on various tumor cells. Beside conventional sialyl-Lewis^x^, the expression of its main ligand sialyl-6-sulfo Lewis^x^ is also present in human colorectal cancer; however, it is preferentially expressed in the nonmalignant colonic epithelia rather than cancer cells [124]. Sialofucosylated podocalyxin, which can bind L- and E-selectin on host cells, is upregulated in a number of cancers including breast, colon, and pancreas malignancies. Hence, the specific depletion of this molecule from the cell surface significantly interferes with selectin-dependent cancer cell-host cell interactions [125]. Similarly to P-selectin, heparin effectively blocked L-selectin preventing leukocyte-endothelial interactions at sites of intravascular arrest [122].

### 4.4. Expression of PSGL-1 on Solid Tumor Cells

PSGL-1, the main counter receptor of selectins, is highly involved not only in inflammation and thrombosis [126, 127], but also in solid tumor progression. Apart from its constitutive presence on normal leukocytes, it is functionally expressed on the surface of tumor cells, such as human prostate carcinoma [128]. The knockdown of PSGL-1 from malignant cells resulted in a significantly reduced aggregate formation between activated platelets and lung cancer cells [129]. Others also claimed that PSGL-1 with CD44 mediated metastasis formation in SCLC cells [95]. Tumor cells produce microparticles at higher quantity that express active tissue factor and PSGL-1. Based on an animal model by Thomas et al., pancreatic and lung cancer cell-derived microparticles bearing PSGL-1 accumulated at the site of injury and played a role in thrombus formation by binding to P-selectin in mice developing a tumor [130].

### 4.5. Selectin Expression on Cancer Cells

Multiple gene defects in tumor cells are generated resulting in phenotypic changes. These “mimicries” are characterized with the reactivation of endothelial or platelet specific genes leading to the expression of E-selectin [131], *α*IIb*β*3 integrin, and thrombin receptor [132–134]. Along this line, selectins are rarely expressed on certain cancer cells to exploit cellular interactions for seeding metastasis. P-selectin was described on metastatic pancreatic tumor cell line, and its expression could be further induced by thrombin stimulation [135].

**Figure 6:**
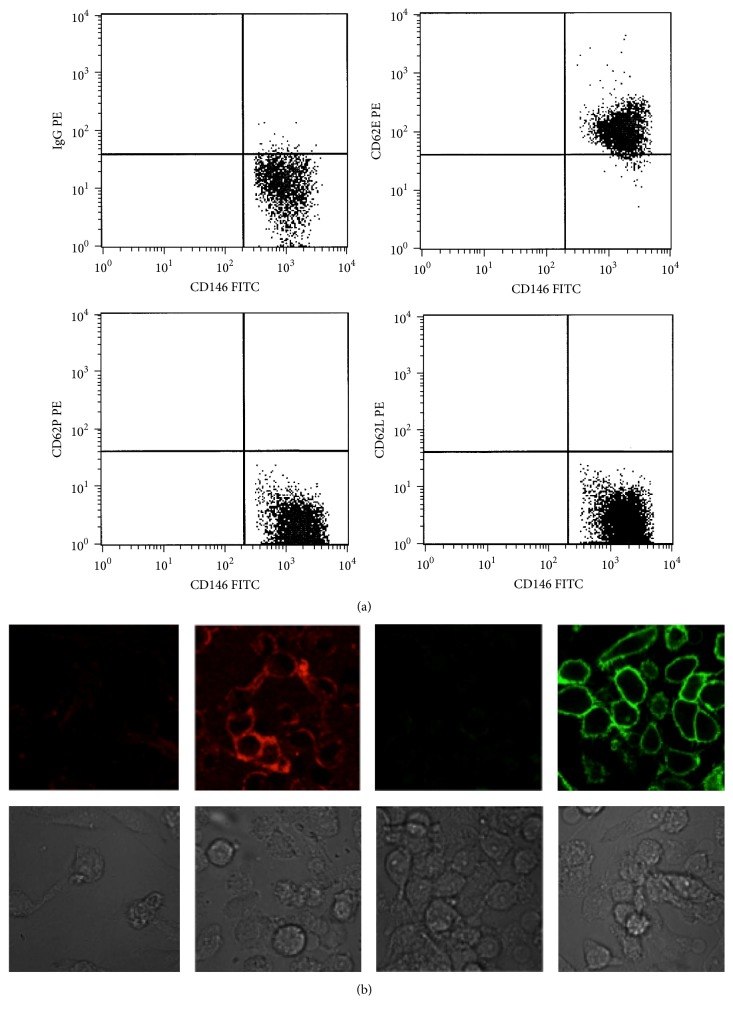
To determine whether any type of selectins may be expressed on cell surface on M35/01 human melanoma cancer cells, flow cytometry (FACSCalibur, Becton Dickinson) was first assessed: (a) Cells were gated based on their CD146-FITC positivity (CD146/MUC18 is an adhesion molecule on melanoma cells [35]) and SSC characteristics (data not shown). These malignant cells showed a substantial E-selectin expression with monoclonal anti-E-selectin antibody but were negative for surface L- and P-selectin. To confirm these results, CLSM (Olympus IX 81 with Fluoview FV 1000) was also used to observe E-selectin (red) and CD146 (green) positivities. (b) IgG-PE and IgG-FITC antibodies were applied to exclude the background staining. Transmission photos (grey) were used to visualize the cells.

**Figure 7:**
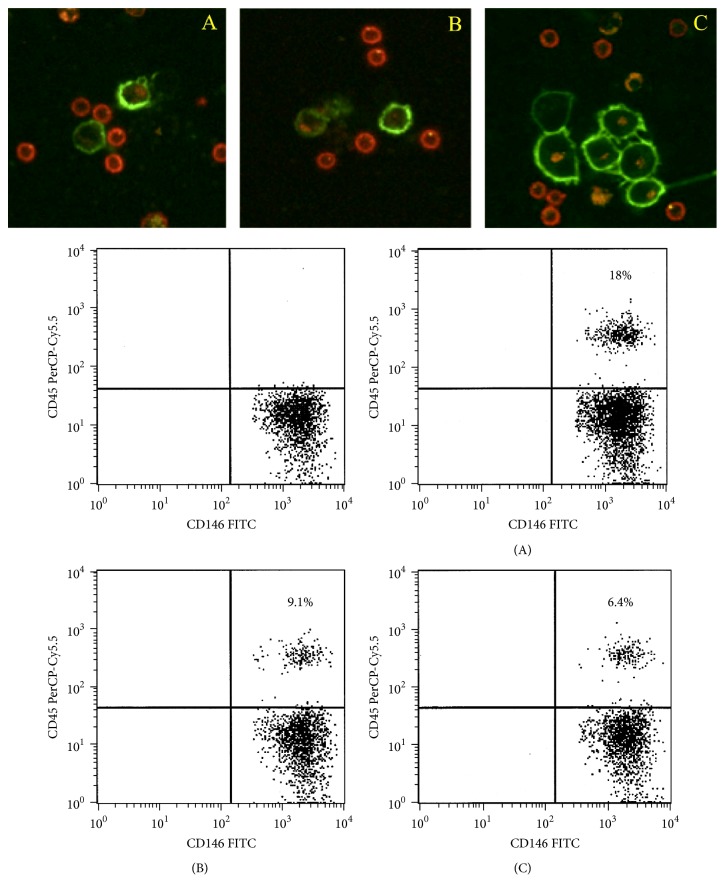
During functional testing of E-selectin on M35/01 melanoma cells by CLSM, these cells were incubated with normal PBMCs for at least 4 hours in the absence (A) and presence of anti-PSGL-1 (B) (clone KPL-1) or anti-E-selectin (C) (clone P2H3) blocking antibodies (BD). Tumor cells were stained with anti-CD146-FITC (green), while anti-CD45-PE (red) (BD) was used for leukocytes to detect heterotypic cellular aggregates. A significantly decreased binding of PBMCs to cancer cells was seen with both blocking antibodies (B, C) suggesting the development of cell-cell interactions via E-selectin and PSGL-1 (A). Representative photos of all conditions are demonstrated. Flow cytometry analysis showed a similar tendency in the ratio of bound PBMCs to cancer cells with (B, C) or without blocking antibodies (A). Double positive events are expressed in % in the dot plots.

Our group has also investigated the expression of selectin(s) on a previously characterized aggressive human melanoma cell line (M35/01) [136]. Based on its endothelial mimicry phenotype, selectin expression was analyzed by flow cytometry and confocal laser scanning microscopy (CLSM). We found that M35/01 melanoma cells showed a substantial E-selectin expression but were negative for surface L- and P-selectin (Figure 6). We then studied if this receptor was functional for the interaction with isolated normal peripheral blood mononuclear cells (PBMCs). For this purpose, PBMCs were added to the tumor cell culture in the absence and presence of blocking antibodies against E-selectin on cancer cells and/or PSGL-1 expressed on PBMCs. The alteration in the attachment of these cells was followed by flow cytometry via measuring CD45 positivity of PBMCs in the gate of melanoma cell population. By CLSM, we visualized the ratio of cellular interactions between the melanoma cells and leukocytes. A significantly decreased binding of PBMCs to cancer cells was seen by both blocking antibodies suggesting the binding of cancer cells to normal leukocytes via E-selectin/PSGL-1 pairing (Figure 7). These data are in accordance with former results on the role of PSGL-1 in tethering leukocytes to E-selectin under flow conditions [137].

### 4.6. The Selectin-PSGL-1 Axis in Hematological Malignancies

According to the literature, selectins and their ligands may also participate in the progression of hematological malignancies. In chronic myeloid leukemia (CML), these receptors are required for homing and engraftment of BCR-ABL1+ leukemic stem cells in the bone marrow niche, since deficiency of E-selectin and L-selectin in the bone marrow endothelium of mice significantly reduced the engraftment of BCR-ABL1-expressing stem cells, while P-selectin was not required [138]. These results establish that BCR-ABL1+ leukemic stem cells rely to a greater extent on selectins and their ligands for homing and engraftment than do normal stem cells and may be beneficial in autologous transplantation for CML [138]. The most frequently observed karyotypic abnormality in acute myeloid leukemia (AML) is the 8 : 21 translocation resulting in the formation of the RUNX1/ETO oncoprotein that suppresses the expression of PSGL-1 on hematopoietic progenitor cells and deregulates other genes involved in differentiation and proliferation. These alterations contribute to impaired adhesive behavior of t(8,21)+ AML cells and may partially explain a favorable response to chemotherapy with a better prognosis [139]. Similarly, PSGL-1 copy number was considered as a biomarker to differentiate different types of AML [140]. In multiple myeloma, PSGL-1 is highly expressed on the myeloma cells and regulates their homing into bone marrow microenvironment [141]. However, the mobilization of mature myeloid cells and their precursors from the bone marrow is also mediated by PSGL-1 via the interaction of leukocytes with endothelial or stromal cells [142]. Finally, function of PSGL-1 is associated with hematogenous metastasis of lymphomas as the downregulation of its expression in metastatic lymphoid cells resulted in a significant reduction of liver and spleen colonization in a dose-dependent manner [143].

### 4.7. Function of Neutrophil Extracellular Traps in Thrombosis and Cancer via P-Selectin

NETs are released from activated neutrophils comprising DNA fibers with histones and various granular proteases [144]. NETs were first identified as a host defense mechanism against bacteria. In activation of neutrophils by pathogens or cytokines, histone H3 becomes hypercitrullinated that leads to chromatin decondensation [145]. Increased NET formation is typical not only in severe infections, but also in thrombotic complications as platelets become activated by NETs [146], and induces thrombin generation and tissue factor expression [147]. In turn, platelets can trigger NETosis via thromboxane A2 release [148]. Solid tumors and leukemias may produce G-CSF that primes more neutrophils for further NET generation [149]. NETs accumulate at the site of neutrophil accumulation and influence cancer environment causing necrotic areas within the tumor being advantageous for tumor growth [150]. Notably, NETs also promote cancer-associated venous thrombosis and arterial microthrombosis as in ischemic stroke [151, 152]. To date, one report is available about the direct association of selectin function and NET formation [153]. Accordingly, surface P-selectin on thrombin activated platelets as well as its soluble form and neutrophil PSGL-1 interaction promotes NETosis [153]. The release of histone was found to induce the neutrophil-endothelium interactions in the muscle microcirculation through P-selectin/PSGL-1 pairing; hence this histone-dependent inflammatory process may be involved after NET generation as well [154]. However, the role of other types of selectin with their ligands in NET formation is still being defined. Several other questions may also arise in terms of NETs, for example, whether neutrophils participating in circulating heterotypic aggregates with platelets may produce more NETs via P-selectin.

### 4.8. Mucin Associated Abnormal Coagulation at Cancer Progression

Selectins also contribute to the development of coagulation disorders that are often detected in cancer subjects [155]. Particularly mucinous carcinomas expressing high level of mucins trigger platelet-rich microthrombi formation that is accompanied with cancer progression [155]. Furthermore, carcinoma mucins promote reciprocal activation of platelets and neutrophils requiring P- and L-selectin but not thrombin in a murine model of Trousseau syndrome [156]. Overall, mucin-selectin interaction has been implicated as one of the potential mechanisms in the frequent development of venous thrombosis in pancreatic cancer [157].

## 5. Conclusion

In this review, we have summarized the central function of selectins and their ligands as key mediators in a number of cellular events during development of thrombotic and malignant conditions. Since these receptors are major contributors to the pathological processes, they represent diagnostic biomarkers and ideal targets for intervention of thrombosis and cancer. As only a limited number of safe specific drugs against selectin ligands are available in humans, future studies are required to investigate more details of cellular selectin-mediated interactions discussed above.

## Figures and Tables

**Table 1:** Characteristics of various selectins.

	Cellular expression	Protein ligands	Rolling velocity of leukocytes
P-selectin (GMP-140, PADGEM, CD62P)	Activated endothelium and platelets	PSGL-1 CD24	Slow

E-Selectin (ELAM-1, CD62E)	Activated endothelium	PSGL-1 ESL-1 L-Selectin Podocalyxin	Slow

L-Selectin (MEL-14, CD62L)	Constitutive expression on leukocytes	PSGL-1 GlyCAM-1 MAdCAM-1 CD-34 Podocalyxin	Fast

## References

[1] Johnston G. I., Cook R. G., McEver R. P. (1989). Cloning of GMP-140, a granule membrane protein of platelets and endothelium: sequence similarity to proteins involved in cell adhesion and inflammation. *Cell*.

[2] Ley K. (2003). The role of selectins in inflammation and disease. *Trends in Molecular Medicine*.

[3] McEver R. P., Martin M. N. (1984). A monoclonal antibody to a membrane glycoprotein binds only to activated platelets. *Journal of Biological Chemistry*.

[4] Stenberg P. E., McEver R. P., Shuman M. A., Jacques Y. V., Bainton D. F. (1985). A platelet alpha-granule membrane protein (GMP-140) is expressed on the plasma membrane after activation. *Journal of Cell Biology*.

[5] Hsu-Lin S.-C., Berman C. L., Furie B. C., August D., Furie B. (1984). A platelet membrane protein expressed during platelet activation and secretion. *Journal of Biological Chemistry*.

[6] Larsen E., Celi A., Gilbert G. E. (1989). PADGEM protein: a receptor that mediates the interaction of activated platelets with neutrophils and monocytes. *Cell*.

[7] McEver R. P., Beckstead J. H., Moore K. L., Marshall-Carlson L., Bainton D. F. (1989). GMP-140, a platelet *α*–granule membrane protein, is also synthesized by vascular endothelial cells and is localized in Weibel–Palade bodies. *The Journal of Clinical Investigation*.

[8] Bevilacqua M. P., Stengelin S., Gimbrone M. A., Seed B. (1989). Endothelial leukocyte adhesion molecule 1: an inducible receptor for neutrophils related to complement regulatory proteins and lectins. *Science*.

[9] Gallatin W. M., Weissman I. L., Butcher E. C. (1983). A cell–urface molecule involved in organ–specific homing of lymphocytes. *Nature*.

[10] Tedder T. F., Isdaacs C. M., Ernst T. J., Demetri G. D., Adler D. A., Disteche C. M. (1989). Isolation and chromosomal localization of cDNAs encoding a novel human lymphocyte cell surface molecule, LAM-1. Homology with the mouse lymphocyte homing receptor and other human adhesion proteins. *Journal of Experimental Medicine*.

[11] Au A. E., Josefsson E. C. (2016). Regulation of platelet membrane protein shedding in health and disease. *Platelets*.

[12] Dole V. S., Bergmeier W., Patten I. S., Hirahashi J., Mayadas T. N., Wagner D. D. (2007). PSGL-1 regulates platelet P-selectin-mediated endothelial activation and shedding of P-selectin from activated platelets. *Thrombosis and Haemostasis*.

[13] Ishiwata N., Takio K., Katayama M. (1994). Alternatively spliced isoform of P-selectin is present in vivo as a soluble molecule. *Journal of Biological Chemistry*.

[14] Setiadi H., Disdier M., Green S. A., Canfield W. M., McEver R. P. (1995). Residues throughout the cytoplasmic domain affect the internalization efficiency of P-selectin. *Journal of Biological Chemistry*.

[15] Setiadi H., Sedgewick G., Erlandsen S. L., McEver R. P. (1998). Interactions of the cytoplasmic domain of P-selectin with clathrin- coated pits enhance leukocyte adhesion under flow. *Journal of Cell Biology*.

[16] Hattori R., Hamilton K. K., Fugate R. D., McEver R. P., Sims P. J. (1989). Stimulated secretion of endothelial von Willebrand factor is accompanied by rapid redistribution to the cell surface of the intracellular granule membrane protein GMP-140. *Journal of Biological Chemistry*.

[17] Schlüter T., Knauth P., Wald S., Boland S., Bohnensack R. (2009). Numb3 is an endocytosis adaptor for the inflammatory marker P-selectin. *Biochemical and Biophysical Research Communications*.

[18] Setiadi H., McEver R. P. (2008). Clustering endothelial E-selectin in clathrin-coated pits and lipid rafts enhances leukocyte adhesion under flow. *Blood*.

[19] Moore K. L., Varki A., McEver R. G. (1991). GMP-140 binds to a glycoprotein receptor on human neutrophils: evidence for a lectin-like interaction. *Journal of Cell Biology*.

[20] Vachino G., Chang X.-J., Veldman G. M. (1995). P-selectin glycoprotein ligand-1 is the major counter–receptor for P- selectin on stimulated T cells and is widely distributed in non–functional form on many lymphocytic cells. *Journal of Biological Chemistry*.

[21] Ushiyama S., Laue T. M., Moore K. L., Erickson H. P., McEver R. P. (1993). Structural and functional characterization of monomeric soluble P-selectin and comparison with membrane P-selectin. *Journal of Biological Chemistry*.

[22] Laszik Z., Jansen P. J., Cummings R. D., Tedder T. F., McEver R. P., Moore K. L. (1996). P-selectin glycoprotein ligand-1 is broadly expressed in cells of myeloid, lymphoid, and dendritic lineage and in some nonhematopoietic cells. *Blood*.

[23] Nagy Jr. B., Miszti-Blasius K., Kerényi A., Clemetson K. J., Kappelmayer J. (2012). Potential therapeutic targeting of platelet-mediated cellular interactions in atherosclerosis and inflammation. *Current Medicinal Chemistry*.

[24] Afshar-Kharghan V., Diz-Küçükkaya R., Ludwig E. H., Marian A. J., López J. A. (2001). Human polymorphism of P-selectin glycoprotein ligand 1 attributable to variable numbers of tandem decameric repeats in the mucinlike region. *Blood*.

[25] Roldán V., González-Conejero R., Marín F., Pineda J., Vicente V., Corral J. (2004). Short alleles of P-selectin glycoprotein ligand-1 protect against premature myocardial infarction. *American Heart Journal*.

[26] Herrmann S.-M., Ricard S., Nicaud V. (1998). The P-selectin gene is highly polymorphic: reduced frequency of the Pro715 allele carriers in patients with myocardial infarction. *Human Molecular Genetics*.

[27] Barbaux S. C., Blankenberg S., Rupprecht H. J. (2001). Association between P-selectin gene polymorphisms and soluble P-selectin levels and their relation to coronary artery disease. *Arteriosclerosis, Thrombosis, and Vascular Biology*.

[28] Carter A. M., Anagnostopoulou K., Mansfield M. W., Grant P. J. (2003). Soluble P-selectin levels, P-selectin polymorphisms and cardiovascular disease. *Journal of Thrombosis and Haemostasis*.

[29] Nagy B., Csongrádi E., Bhattoa H. P. (2007). Investigation of Thr715Pro P-selectin gene polymorphism and soluble P-selectin levels in type 2 diabetes mellitus. *Thrombosis and Haemostasis*.

[30] Csongrádi E., Nagy B., Fulop T. (2011). Increased levels of platelet activation markers are positively associated with carotid wall thickness and other atherosclerotic risk factors in obese patients. *Thrombosis and Haemostasis*.

[31] Undas A., Tracz W., Siudak Z. (2009). Thr715Pro P-selectin polymorphism and P-selectin release in blood obtained from the bleeding time wounds in patients with deep–vein thrombosis. *Thrombosis Research*.

[32] Revelle B. M., Scott D., Beck P. J. (1996). Single amino acid residues in the E- and P-selectin epidermal growth factor domains can determine carbohydrate binding specificity. *The Journal of Biological Chemistry*.

[33] Wenzel K., Stahn R., Speer A. (1999). Functional characterization of atherosclerosis-associated Ser128Arg and Leu554Phe E-selectin mutations. *Biological Chemistry*.

[34] Berardi C., Larson N. B., Decker P. A. (2015). Multi-ethnic analysis reveals soluble l-selectin may be post-transcriptionally regulated by 3′UTR polymorphism: the Multi-Ethnic Study of Atherosclerosis (MESA). *Human Genetics*.

[35] Wang Z., Yan X. (2013). CD146, a multi-functional molecule beyond adhesion. *Cancer Letters*.

[36] Wild M. K., Lühn K., Marquardt T., Vestweber D. (2002). Leukocyte adhesion deficiency II: therapy and genetic defect. *Cells Tissues Organs*.

[37] Malý P., Thall A. D., Petryniak B. (1996). The *α*(1,3)fucosyltransferase Fuc-TVII controls leukocyte trafficking through an essential role in L-, E-, and P-selectin ligand biosynthesis. *Cell*.

[38] Ramachandran V., Yago T., Epperson T. K. (2001). Dimerization of a selectin and its ligand stabilizes cell rolling and enhances tether strength in shear flow. *Proceedings of the National Academy of Sciences of the United States of America*.

[39] Miner J. J., Xia L., Yago T. (2008). Separable requirements for cytoplasmic domain of PSGL-1 in leukocyte rolling and signaling under flow. *Blood*.

[40] Koenig A., Norgard-Sumnicht K., Linhardt R., Varki A. (1998). Differential interactions of heparin and heparan sulfate glycosaminoglycans with the selectins: implications for the use of unfractionated and low molecular weight heparins as therapeutic agents. *Journal of Clinical Investigation*.

[41] Wang L., Brown J. R., Varki A., Esko J. D. (2002). Heparin's anti-inflammatory effects require glucosamine 6-O-sulfation and are mediated by blockade of L- and P-selectins. *Journal of Clinical Investigation*.

[42] Takeishi N., Imai Y., Ishida S., Omori T., Kamm R. D., Ishikawa T. (2016). Cell adhesion during bullet motion in capillaries. *American Journal of Physiology–Heart and Circulatory Physiology*.

[43] Marki A., Esko J. D., Pries A. R., Ley K. (2015). Role of the endothelial surface layer in neutrophil recruitment. *Journal of Leukocyte Biology*.

[44] Sreeramkumar V., Adrover J. M., Ballesteros I. (2014). Neutrophils scan for activated platelets to initiate inflammation. *Science*.

[45] Zuchtriegel G., Uhl B., Puhr-Westerheide D. (2016). Platelets guide leukocytes to their sites of extravasation. *PLoS Biology*.

[46] Rossaint J., Kühne K., Skupski J. (2016). Directed transport of neutrophil–derived extracellular vesicles enables platelet–mediated innate immune response. *Nature Communications*.

[47] Posner M. G., Upadhyay A., Abubaker A. A. (2016). Extracellular fibrinogen–binding protein (Efb) from staphylococcus aureus inhibits the formation of platelet–leukocyte complexes. *Journal of Biological Chemistry*.

[48] Kappelmayer J., Nagy B., Miszti-Blasius K., Hevessy Z., Setiadi H. (2004). The emerging value of P-selection as a disease marker. *Clinical Chemistry and Laboratory Medicine*.

[49] Michelson A. D., Barnard M. R., Krueger L. A., Valeri C. R., Furman M. I. (2001). Circulating monocyte-platelet aggregates are a more sensitive marker of in vivo platelet activation than platelet surface P-selectin: studies in baboons, human coronary intervention, and human acute myocardial infarction. *Circulation*.

[50] Blann A. D., Lip G. Y. H., Beevers D. G., McCollum C. N. (1997). Soluble P-selectin in atherosclerosis: a comparison with endothelial cell and platelet markers. *Thrombosis and Haemostasis*.

[51] Fijnheer R., Frijns C. J. M., Korteweg J. (1997). The origin of P-selectin as a circulating plasma protein. *Thrombosis and Haemostasis*.

[52] Chen Y., Lin Y., Lin H. (2016). Regular plateletpheresis increased basal concentrations of soluble P-selectin in healthy donors: possible involvement of endothelial cell activation?. *Clinica Chimica Acta*.

[53] Miyabe Y., Kim N. D., Miyabe C., Luster A. D. (2016). Studying neutrophil migration in vivo using adoptive cell transfer. *Methods in Molecular Biology*.

[54] Palabrica T., Lobb R., Furie B. C. (1992). Leukocyte accumulation promoting fibrin deposition is mediated in vivo by P-selectin on adherent platelets. *Nature*.

[55] Celi A., Pellegrini G., Lorenzet R. (1994). P-selectin induces the expression of tissue factor on monocytes. *Proceedings of the National Academy of Sciences of the United States of America*.

[56] Furie B., Furie B. C. (2004). Role of platelet P-selectin and microparticle PSGL-1 in thrombus formation. *Trends in Molecular Medicine*.

[57] Falati S., Liu Q., Gross P. (2003). Accumulation of tissue factor into developing thrombi in vivo is dependent upon microparticle P-selectin glycoprotein ligand 1 and platelet P-selectin. *Journal of Experimental Medicine*.

[58] Slotta J. E., Braun O. O., Menger M. D., Thorlacius H. (2009). Capture of platelets to the endothelium of the femoral vein is mediated by CD62P and CD162. *Platelets*.

[59] Frenette P. S., Denis C. V., Weiss L. (2000). P-selectin glycoprotein ligand 1 (PSGL-1) is expressed on platelets and can mediate platelet-endothelial interactions in vivo. *Journal of Experimental Medicine*.

[60] Kim K. H., Barazia A., Cho J. (2013). Real–time imaging of heterotypic platelet–neutrophil interactions on the activated endothelium during vascular inflammation and thrombus formation in live mice. *Journal of visualized experiments*.

[61] Del Conde I., Nabi F., Tonda R., Thiagarajan P., López J. A., Kleiman N. S. (2005). Effect of P-selectin on phosphatidylserine exposure and surface–dependent thrombin generation on monocytes. *Arteriosclerosis, Thrombosis, and Vascular Biology*.

[62] Del Conde I., Shrimpton C. N., Thiagarajan P., López J. A. (2005). Tissue–factor–bearing microvesicles arise from lipid rafts and fuse with activated platelets to initiate coagulation. *Blood*.

[63] Théorêt J.-F., Yacoub D., Hachem A., Gillis M.-A., Merhi Y. (2011). P-selectin ligation induces platelet activation and enhances microaggregate and thrombus formation. *Thrombosis Research*.

[64] Steeber D. A., Engel P., Miller A. S., Sheetz M. P., Tedder T. F. (1997). Ligation of L-selectin through conserved regions within the lectin domain activates signal transduction pathways and integrin function in human, mouse, and rat leukocytes. *Journal of Immunology*.

[65] Ozaki Y., Imanishi T., Teraguchi I. (2014). Association between P-selectin glycoprotein ligand-1 and pathogenesis in acute coronary syndrome assessed by optical coherence tomography. *Atherosclerosis*.

[66] Miszti-Blasius K., Debreceni I. B., Felszeghy S., Dezso B., Kappelmayer J. (2011). Lack of P-selectin glycoprotein ligand-1 protects mice from thrombosis after collagen/epinephrine challenge. *Thrombosis Research*.

[67] Wang H., Kleiman K., Wang J., Luo W., Guo C., Eitzman D. T. (2015). Deficiency of P-selectin glycoprotein ligand-1 is protective against the prothrombotic effects of interleukin-1*β*. *Journal of Thrombosis and Haemostasis*.

[68] Wang H., Knight J. S., Hodgin J. B. (2016). Psgl-1 deficiency is protective against stroke in a murine model of lupus. *Scientific Reports*.

[69] Hrachovinová I., Cambien B., Hafezi-Moghadam A. (2003). Interaction of P-selectin and PSGL-1 generates microparticles that correct hemostasis in a mouse model of hemophilia A. *Nature Medicine*.

[70] Hubert L., Darbousset R., Panicot-Dubois L. (2014). Neutrophils recruit and activate human endothelial colony-forming cells at the site of vessel injury via P-selectin glycoprotein ligand-1 and L-selectin. *Journal of Thrombosis and Haemostasis*.

[71] Läubli H., Borsig L. (2010). Selectins promote tumor metastasis. *Seminars in Cancer Biology*.

[72] Glavey S. V., Huynh D., Reagan M. R. (2015). The cancer glycome: carbohydrates as mediators of metastasis. *Blood Reviews*.

[73] Kannagi R., Izawa M., Koike T., Miyazaki K., Kimura N. (2004). Carbohydrate-mediated cell adhesion in cancer metastasis and angiogenesis. *Cancer Science*.

[74] Ogawa J., Inoue H., Koide S. (1996). Expression of alpha-1, 3-fucosyltransferase type IV and VII genes is related to poor prognosis in lung cancer. *Cancer Res*.

[75] Hoos A., Protsyuk D., Borsig L. (2014). Metastatic growth progression caused by PSGL-1- Mediated recruitment of monocytes to metastatic sites. *Cancer Research*.

[76] Mannori G., Cecconi O., Hanasaki K. (1995). Differential colon cancer cell adhesion to E-, P-, and L-selectin: role of mucin–type glycoproteins. *Cancer Research*.

[77] Chen S.-H., Dallas M. R., Balzer E. M., Konstantopoulos K. (2012). Mucin 16 is a functional selectin ligand on pancreatic cancer cells. *FASEB Journal*.

[78] Borsig L., Wong R., Feramisco J., Nadeau D. R., Varki N. M., Varki A. (2001). Heparin and cancer revisited: mechanistic connections involving platelets, P-selectin, carcinoma mucins, and tumor metastasis. *Proceedings of the National Academy of Sciences of the United States of America*.

[79] Gout S., Tremblay P.-L., Huot J. (2008). Selectins and selectin ligands in extravasation of cancer cells and organ selectivity of metastasis. *Clinical and Experimental Metastasis*.

[80] Läubli H., Borsig L. (2010). Selectins as mediators of lung metastasis. *Cancer Microenvironment*.

[81] Nash G. F., Turner L. F., Scully M. F., Kakkar A. K. (2002). Platelets and cancer. *Lancet Oncology*.

[82] Kaplan R. N., Riba R. D., Zacharoulis S. (2005). VEGFR1-positive haematopoietic bone marrow progenitors initiate the pre-metastatic niche. *Nature*.

[83] Hiratsuka S., Watanabe A., Aburatani H., Maru Y. (2006). Tumour–mediated upregulation of chemoattractants and recruitment of myeloid cells predetermines lung metastasis. *Nature Cell Biology*.

[84] Gakhar G., Navarro V. N., Jurish M. (2013). Circulating tumor cells from prostate cancer patients interact with E-selectin under physiologic blood flow. *PLoS ONE*.

[85] Kang S.-A., Hasan N., Mann A. P. (2015). Blocking the adhesion cascade at the premetastatic niche for prevention of breast cancer metastasis. *Molecular Therapy*.

[86] Shi H., Zhang J., Han X. (2017). Recruited monocytic myeloid–derived suppressor cells promote the arrest of tumor cells in the premetastatic niche through an IL-1*β*–mediated increase in E-selectin expression. *International Journal of Cancer*.

[87] Läubli H., Spanaus K.-S., Borsig L. (2009). Selectin–mediated activation of endothelial cells induces expression of CCL5 and promotes metastasis through recruitment of monocytes. *Blood*.

[88] Dong C., Slattery M. J., Liang S., Peng H. H. (2005). Melanoma cell extravasation under flow conditions is modulated by leukocytes and endogenously produced interleukin 8. *Mol Cell Biomech*.

[89] Mayadas T. N., Johnson R. C., Rayburn H., Hynes R. O., Wagner D. D. (1993). Leukocyte rolling and extravasation are severely compromised in P selectin–deficient mice. *Cell*.

[90] Aruffo A., Dietsch M. T., Wan H., Hellström K. E., Hellström I. (1992). Granule membrane protein 140 (GMP140) binds to carcinomas and carcinoma–derived cell lines. *Proceedings of the National Academy of Sciences of the United States of America*.

[91] Goetz D. J., Ding H., Atkinson W. J. (1996). A human colon carcinoma cell line exhibits adhesive interactions with P- selectin under fluid flow via a PSGL-1-independent mechanism. *American Journal of Pathology*.

[92] Pottratz S. T., Hall T. D., Scribner W. M., Jayaram H. N., Natarajan V. (1996). P-selectin–mediated attachment of small cell lung carcinoma to endothelial cells. *American Journal of Physiology–Lung Cellular and Molecular Physiology*.

[93] Kim Y. J., Borsig L., Varki N. M., Varki A. (1998). P-selectin deficiency attenuates tumor growth and metastasis. *Proceedings of the National Academy of Sciences of the United States of America*.

[94] Borsig L., Wong R., Hynes R. O., Varki N. M., Varki A. (2002). Synergistic effects of L- and P-selectin in facilitating tumor metastasis can involve non–mucin ligands and implicate leukocytes as enhancers of metastasis. *Proceedings of the National Academy of Sciences of the United States of America*.

[95] Heidemann F., Schildt A., Schmid K. (2014). Selectins mediate small cell lung cancer systemic metastasis. *PLoS ONE*.

[96] Borsig L. (2008). The role of platelet activation in tumor metastasis. *Expert Review of Anticancer Therapy*.

[97] Karpatkin S., Pearlstein E., Ambrogio C., Coller B. S. (1988). Role of adhesive proteins in platelet tumor interaction in vitro and metastasis formation in vivo. *Journal of Clinical Investigation*.

[98] Kaytes P. S., Geng J.-G. (1998). P-selectin mediates adhesion of the human melanoma cell line NKI-4: identification of glycoprotein ligands. *Biochemistry*.

[99] Ma Y.-Q., Geng J.-G. (2000). Heparan sulfate-like proteoglycans mediate adhesion of human malignant melanoma A375 cells to P-selectin under flow. *Journal of Immunology*.

[100] Ma Y.-Q., Geng J.-G. (2002). Obligatory requirement of sulfation for P-selectin binding to human salivary gland carcinoma Acc-M cells and breast carcinoma ZR-75-30 cells. *Journal of Immunology*.

[101] Li L., Short H. J., Qian K.-X., Elhammer A. P., Geng J.-G. (2001). Characterization of glycoprotein ligands for P-selectin on a human small cell lung cancer cell line NCI-H345. *Biochemical and Biophysical Research Communications*.

[102] Aigner S., Sthoeger Z. M., Fogel M. (1997). CD24, a mucin-type glycoprotein, is a ligand for P-selectin on human tumor cells. *Blood*.

[103] Nieswandt B., Hafner M., Echtenacher B., Männel D. N. (1999). Lysis of tumor cells by natural killer cells in mice is impeded by platelets. *Cancer Research*.

[104] Ludwig R. J., Boehme B., Podda M. (2004). Endothelial P-Selectin as a target of heparin action in experimental melanoma lung metastasis. *Cancer Research*.

[105] Balkwill F., Mantovani A. (2001). Inflammation and cancer: back to virchow?. *Lancet*.

[106] Nasti T. H., Bullard D. C., Yusuf N. (2015). P-selectin enhances growth and metastasis of mouse mammary tumors by promoting regulatory T cell infiltration into the tumors. *Life Sciences*.

[107] Schlesinger M., Roblek M., Ortmann K. (2014). The role of VLA-4 binding for experimental melanoma metastasis and its inhibition by heparin. *Thrombosis Research*.

[108] Garcia J., Callewaert N., Borsig L. (2007). P-selectin mediates metastatic progression through binding to sulfatides on tumor cells. *Glycobiology*.

[109] Brodt P., Fallavollita L., Bresalier R. S., Meterissian S., Norton C. R., Wolitzky B. A. (1997). Liver endothelial E-selectin mediates carcinoma cell adhesion and promotes liver metastasis. *International Journal of Cancer*.

[110] Mannori G., Santoro D., Carter L., Corless C., Nelson R. M., Bevilacqua M. P. (1997). Inhibition of colon carcinoma cell lung colony formation by a soluble form of E-selectin. *American Journal of Pathology*.

[111] Aychek T., Miller K., Sagi-Assif O. (2008). E-selectin regulates gene expression in metastatic colorectal carcinoma cells and enhances HMGB1 release. *International Journal of Cancer*.

[112] Tremblay P.-L., Auger F. A., Huot J. (2006). Regulation of transendothelial migration of colon cancer cells by E-selectin-mediated activation of p38 and ERK MAP kinases. *Oncogene*.

[113] Burdick M. M., Chu J. T., Godar S., Sackstein R. (2006). HCELL is the major E- and L-selectin ligand expressed on LS174T colon carcinoma cells. *Journal of Biological Chemistry*.

[114] Gout S., Morin C., Houle F., Huot J. (2006). Death receptor-3, a new E-selectin counter-receptor that confers migration and survival advantages to colon carcinoma cells by triggering p38 and ERK MAPK activation. *Cancer Research*.

[115] Tomlinson J., Wang J. L., Barsky S. H., Lee M. C., Bischoff J., Nguyen M. (2000). Human colon cancer cells express multiple glycoprotein ligands for E-selectin. *International Journal of Oncology*.

[116] Shirure V. S., Reynolds N. M., Burdick M. M. (2012). Mac-2 binding protein is a novel E-selectin ligand expressed by breast cancer cells. *PLoS ONE*.

[117] Stevenson J. L., Varki A., Borsig L. (2007). Heparin attenuates metastasis mainly due to inhibition of P- and L-selectin, but non–anticoagulant heparins can have additional effects. *Thrombosis Research*.

[118] Park K., Ki Lee S., Hyun Son D. (2007). The attenuation of experimental lung metastasis by a bile acid acylated-heparin derivative. *Biomaterials*.

[119] Hosono J., Narita T., Kimura N. (1998). Involvement of adhesion molecules in metastasis of SW1990, human pancreatic cancer cells. *Journal of Surgical Oncology*.

[120] Insug O., Otvos L., Kieber-Emmons T., Blaszczyk-Thurin M. (2002). Role of SA-Lea and E-selectin in metastasis assessed with peptide antagonist. *Peptides*.

[121] Qian F., Hanahan D., Weissman I. L. (2001). L-selectin can facilitate metastasis to lymph nodes in a transgenic mouse model of carcinogenesis. *Proceedings of the National Academy of Sciences of the United States of America*.

[122] Läubli H., Stevenson J. L., Varki A., Varki N. M., Borsig L. (2006). L-selectin facilitation of metastasis involves temporal induction of Fut7-dependent ligands at sites of tumor cell arrest. *Cancer Research*.

[123] Yamada M., Yanaba K., Hasegawa M. (2006). Regulation of local and metastatic host–mediated anti–tumour mechanisms by L-selectin and intercellular adhesion molecule-1. *Clinical and Experimental Immunology*.

[124] Izawa M., Kumamoto K., Mitsuoka C. (2000). Expression of sialyl 6-sulfo Lewis X is inversely correlated with conventional sialyl Lewis X expression in human colorectal cancer. *Cancer Research*.

[125] Dallas M. R., Chen S.-H., Streppel M. M., Sharma S., Maitra A., Konstantopoulos K. (2012). Sialofucosylated podocalyxin is a functional E- and L-selectin ligand expressed by metastatic pancreatic cancer cells. *American Journal of Physiology–Cell Physiology*.

[126] McEver R. P., Cummings R. D. (1997). Role of PSGL-1 binding to selectins in leukocyte recruitment. *Journal of Clinical Investigation*.

[127] Vandendries E. R., Furie B. C., Furie B. (2004). Role of P-selectin and PSGL-I in coagulation and thrombosis. *Thrombosis and Haemostasis*.

[128] Dimitroff C. J., Descheny L., Trujillo N. (2005). Identification of leukocyte E-selectin ligands, P-selectin glycoprotein ligand-1 and E-selectin ligand-1, on human metastatic prostate tumor cells. *Cancer Research*.

[129] Gong L., Cai Y., Zhou X., Yang H. (2012). Activated platelets interact with lung cancer cells through P-selectin glycoprotein ligand-1. *Pathology and Oncology Research*.

[130] Thomas G. M., Panicot-Dubois L., Lacroix R., Dignat-George F., Lombardo D., Dubois C. (2009). Cancer cell–erived microparticles bearing P-selectin glycoprotein ligand 1 accelerate thrombus formation in vivo. *Journal of Experimental Medicine*.

[131] Hendrix M. J. C., Seftor E. A., Hess A. R., Seftor R. E. B. (2003). Vasculogenic mimicry and tumour–cell plasticity: lessons from melanoma. *Nature Reviews Cancer*.

[132] Tímár J., Tóvári J., Rásó E., Mészáros L., Bereczky B., Lapis K. (2005). Platelet–mimicry of cancer cells: epiphenomenon with clinical significance. *Oncology*.

[133] Trikha M., Timar J., Lundy S. K. (1997). The high affinity *α*IIb*β*3 integrin is involved in invasion of human melanoma cells. *Cancer Research*.

[134] Tellez C., McCarty M., Ruiz M., Bar-Eli M. (2003). Loss of activator protein-2*α* results in overexpression of protease–activated receptor-1 and correlates with the malignant phenotype of human melanoma. *Journal of Biological Chemistry*.

[135] Iwamura T., Caffrey T. C., Kitamura N., Yamanari H., Setoguchi T., Hollingsworth M. A. (1997). P-selectin expression in a metastatic pancreatic tumor cell line (SUIT- 2). *Cancer Research*.

[136] Treszl A., Ladanyi A., Rakosy Z., Buczko Z., Adany R., Balazs M. (2006). Molecular cytogenetic characterization of a novel cell line established from a superficial spreading melanoma. *Frontiers in Bioscience*.

[137] Xia L., Sperandio M., Yago T. (2002). P-selectin glycoprotein ligand-1-deficient mice have impaired leukocyte tethering to E-selectin under flow. *Journal of Clinical Investigation*.

[138] Krause D. S., Lazarides K., Lewis J. B., Von Andrian U. H., Van Etten R. A. (2014). Selectins and their ligands are required for homing and engraftment of BCR-ABL1^+^ leukemic stem cells in the bone marrow niche. *Blood*.

[139] Ponnusamy K., Kohrs N., Ptasinska A. (2015). RUNX1/ETO blocks selectin–mediated adhesion via epigenetic silencing of PSGL-1. *Oncogenesis*.

[140] Kappelmayer J., Kiss A., Karászi É., Veszprémi A., Jakó J., Kiss C. (2001). Identification of P-selectin glycoprotein ligand-1 as a useful marker in acute myeloid leukaemias. *British Journal of Haematology*.

[141] Azab A. K., Quang P., Azab F. (2012). P-selectin glycoprotein ligand regulates the interaction of multiple myeloma cells with the bone marrow microenvironment. *Blood*.

[142] Miszti-Blasius K., Felszeghy S., Kiss C. (2014). P-selectin glycoprotein ligand-1 deficiency augments G-CSF induced myeloid cell mobilization. *Naunyn-Schmiedeberg's Archives of Pharmacology*.

[143] Raes G., Ghassabeh G. H., Brys L. (2007). The metastatic T-cell hybridoma antigen/P-selectin glycoprotein ligand 1 is required for hematogenous metastasis of lymphomas. *International Journal of Cancer*.

[144] Brinkmann V., Reichard U., Goosmann C. (2004). Neutrophil extracellular traps kill bacteria. *Science*.

[145] Li P., Li M., Lindberg M. R., Kennett M. J., Xiong N., Wang Y. (2010). PAD4 is essential for antibacterial innate immunity mediated by neutrophil extracellular traps. *The Journal of Experimental Medicine*.

[146] Fuchs T. A., Brill A., Duerschmied D. (2010). Extracellular DNA traps promote thrombosis. *Proceedings of the National Academy of Sciences of the United States of America*.

[147] Kambas K., Mitroulis I., Ritis K. (2012). The emerging role of neutrophils in thrombosis–he journey of TF through NETs. *Frontiers in Immunology*.

[148] Caudrillier A., Kessenbrock K., Gilliss B. M. (2012). Platelets induce neutrophil extracellular traps in transfusion–related acute lung injury. *The Journal of Clinical Investigation*.

[149] Demers M., Wagner D. D. (2013). Neutrophil extracellular traps: a new link to cancer–associated thrombosis and potential implications for tumor progression. *OncoImmunology*.

[150] Demers M., Wagner D. D. (2014). NETosis: a new factor in tumor progression and cancer–associated thrombosis. *Seminars in Thrombosis and Hemostasis*.

[151] Demers M., Krause D. S., Schatzberg D. (2012). Cancers predispose neutrophils to release extracellular DNA traps that contribute to cancer–associated thrombosis. *Proceedings of the National Academy of Sciences of the United States of America*.

[152] Thålin C., Demers M., Blomgren B. (2016). NETosis promotes cancer–associated arterial microthrombosis presenting as ischemic stroke with troponin elevation. *Thrombosis Research*.

[153] Etulain J., Martinod K., Wong S. L., Cifuni S. M., Schattner M., Wagner D. D. (2015). P-selectin promotes neutrophil extracellular trap formation in mice. *Blood*.

[154] Puegge J., Wang Y., Roller J. (2016). Adhesive mechanisms of histone–induced neutrophil–endothelium interactions in the muscle microcirculation. *European Surgical Research*.

[155] Wahrenbrock M., Borsig L., Le D., Varki N., Varki A. (2003). Selectin–mucin interactions as a probable molecular explanation for the association of Trousseau syndrome with mucinous adenocarcinomas. *Journal of Clinical Investigation*.

[156] Shao B., Wahrenbrock M. G., Yao L. (2011). Carcinoma mucins trigger reciprocal activation of platelets and neutrophils in a murine model of Trousseau syndrome. *Blood*.

[157] Ansari D., Andersson R., Andrén-Sandberg A. (2015). Pancreatic cancer and thromboembolic disease, 150 years after Trousseau. *Hepatobiliary Surgery and Nutrition*.

